# Serum concentrations of progesterone and prolactin as predictors of success in *in vitro* fertilization: A retrospective cohort study

**DOI:** 10.5935/1518-0557.20220071

**Published:** 2023

**Authors:** Paulina Alejandra Santander-Pérez, Álvaro Pigatto Ceschin, Ana Amélia Bartolamei Ramos, Renato Nisihara, Jaime Kulak Junior

**Affiliations:** 1 Post Graduate Program in Tocogynecology and Women Health, Federal University of Paraná, Curitiba, Brazil; 2 Feliccitá Fertility Institute, Curitiba, Brazil; 3 Department of Medicine, Positivo University, Curitiba, Brazil; 4 Departament of Tocogynecology, Federal University of Paraná, Curitiba, Brazil

**Keywords:** progesterone, prolactin, *in vitro* fertilization, gestation

## Abstract

**Objective:**

The aim of this study is to determine whether the serum concentrations of progesterone and/or prolactin after fresh embryo transfer are associated with pregnancy rates in in vitro fertilization treatment.

**Methods:**

This retrospective cohort study evaluated in vitro fertilization treatments with fresh embryo transfer, which were performed between 2013 and 2019 in a private clinic in Curitiba, Brazil. The serum concentrations of progesterone and prolactin were evaluated by chemiluminescence nine days after oocyte retrieval. The type of progesterone supplementation and pituitary blockage during the cycle of oocyte stimulation were evaluated.

**Results:**

330 fertilization cycles were performed in the 293 studied patients. The mean age of patients was 35.5±4.1years. The most seen isolated infertility factor was endometriosis (24.2% of the cases), while progesterone supplementation was performed intramuscularly in 73.9% of the cases. The progesterone values above 32.1ng/ml at day 9 (D9) were associated with better pregnancy rates. In cycles using antagonist and intramuscular luteal phase supplementation, higher pregnancy rates with progesterone values above 37.83ng/ml were observed. Moreover, prolactin showed no significant association with any of the studied variables.

**Conclusions:**

The serum progesterone concentrations above 32.1ng/ml at D9 that were taken one week before pregnancy testing were associated with successful in vitro fertilization treatment. Furthermore, prolactin showed no significant association with any of the studied variables.

## INTRODUCTION

Infertility affects 15 to 20% of the population of reproductive age (Vander Borght & Wyns, 2018). The Brazilian Institute of Geography and Statistics (IBGE) counted the Brazilian population at 211 million in June 2020. Of these, 52.97% are of reproductive age, accounting for 111,7 million of the population. Thus, 16,765,000 people in Brazil are infertile (IBGE, 2020).

Part of this population will need in vitro fertilization (IVF), a treatment that still has limited access to qualified professionals and is expensive (Inhorn & Patrizio, 2015). Personalized care for these patients during treatment is required. The greater the amount of information that increases the success rates, the better the public will be served, in addition to also benefiting the assistant physician in the clinical practice of IVF.

A good embryo and endometrium, as well as adequate hormone levels, are needed to achieve pregnancy, with progesterone (P4) being a contributing factor. Produced by the corpus luteum, P4 is essential in the second phase of the menstrual cycle (Elgindy *et al*., 2018), which facilitates embryonic fixation (Drakopoulos *et al*., 2019). P4 values depend on the period of the cycle in which they were collected (Taylor *et al*., 2019a). In IVF, several corpora lutea are produced, but ineffectively, making P4 supplementation necessary (Fatemi, 2009).

Prolactin (PRL) plays a role in implantation, maintenance of pregnancy, and lactation. Unlike P4, it does not undergo variations in the menstrual cycle (Taylor *et al*., 2019a). The increase in the serum concentrations of PRL can interfere with the proliferation of granulosa cells, leading to a drop in P4, luteal phase insufficiency, and failure of embryo implantation (Kamel *et al*., 2018). In abortions, a decrease in PRL receptors in the endometrium can be observed, suggesting its importance in maintaining pregnancy (Kim *et al*., 2018).

Some values of hormone dosages are established. On the human chorionic gonadotropin (hCG) day, values above 1.5ng/ml indicate early endometrial luteinization, allowing the choice to freeze the embryos and transfer them in another cycle (Panaino *et al*., 2017). In frozen embryo transfer, P4 values greater than 10 ng/ml on Day 4 (D4) of embryonic evolution are described as a predictor of success (Gaggiotti-Marre *et al*., 2019).

No parameters on the values of the serum concentrations of P4 and PRL at the time between embryo transfer and the performance of beta-hCG were mentioned in the literature. No values for progesterone on D9 after oocyte retrieval, one week before the pregnancy test, were established. Thus, the aim of this study is to evaluate whether the serum dosage of P4 and PRL, performed on D9 after oocyte retrieval and between the embryo transfer and pregnancy test, is related to success in IVF and, if so, determine the cut-off points.

## MATERIAL AND METHODS

### Study design and ethical aspects

This observational, retrospective study analyzed the medical data of patients undergoing IVF with fresh embryo transfer in a private clinic in Curitiba, Brazil. The investigation was conducted in accordance with the Declaration of Helsinki and received a favorable opinion from the ethics committee of the Health Sciences Sector of the Hospital de Clínicas of the Federal University of Paraná (HC-UFPR) on October 2, 2018, under number 2.932.466.

### Samples and data collected

The fresh embryo transfer cycles performed between January 2013 and December 2019 were reviewed. The data collected were age, infertility factors, endometrial thickness on the hCG day, type of pituitary blockage, embryonic quality, number of embryos transferred, types and doses of P4 used in luteal phase support, serum P4 and PRL values collected on D9 after oocyte puncture, and pregnancy symptoms.

The serum concentrations of P4 and PRL of the samples were determined by chemiluminescence with the support of the laboratory of the center where the research was conducted. All cases used the same methodology. P4 and PRL serum concentrations were measured using Architech-Abbott^®^, Beckman Coulter-Access^®^, and Siemens ADVIA Centaur^®^ equipment.

### Inclusion and exclusion criteria

Cases of IVF cycles and fresh embryo transfer were included, with P4 and PRL collection in the morning, nine days after oocyte retrieval. Cycles with own oocytes and fresh embryo transfer; trilaminar endometrium between 07 and 13 mm; transfer of at least one Lucinda Veeck Category A or B cleaved embryo (Veeck, 1999) and/or good quality Gardner blastocysts (Gardner & Schoolcraft, 1999); easy embryo transfer; and the necessary serum dosages for the study were evaluated.

Cases with incomplete data for the purpose of this study, transfer of frozen material, category C/D embryos or poor quality blastocysts, transfers with blood and/or mucus in the catheter, the use of Pozzi or obturator, and embryos retained in the catheter were excluded.

### Statistical analysis

The data was analyzed by frequency and contingency tables. Fisher and chi-square tests were used for the comparison of nominal data, while an unpaired t-test was used for the comparison of numerical data.

Significant data was studied in ROC (receive operator characteristic) curves to establish the relationship between the sensitivity and specificity of the quantitative diagnostic test.

A univariate analysis of these parameters was performed in a logistic regression model. The multivariate analysis observed the relevant criteria among themselves.

The Stata/SE v.14.1 programs (StataCorpLP, USA) and GraphPad Prism for Mac OS version 8.4.3 (San Diego, California, USA) (www.graphpad.com) was used.

## RESULTS

A total of 675 IVF cycles with fresh embryo transfer were evaluated. In accordance with the inclusion and exclusion criteria, 330 cycles performed in 293 patients were studied.

The mean age of the patients was 35.5±4.1 years. The most common causes of infertility were endometriosis in 24.2% of cases; male factor in 20.6%; and ovarian failure and tubal factor in 15.5% and 15.8%, respectively, in addition to the combinations of factors. The endometrial thickness assessed by ultrasound on the day of the trigger was 9.1±1.6 mm.

Pituitary blockage was performed with GnRH (gonadotropin-releasing hormone) analog in 118 cycles (35.8%); GnRH antagonist in 185 cycles (56.1%); and no blockage in 27 cycles (8.2%). P4 supplementation was performed intramuscularly (IM) with 50 mg/day in 73.9% of the cycles. Micronized natural progesterone (800 mg/day) (Utrogestan^®^) was used in 14.6% of the cases. In 11.5% of the cases, vaginal gel (90 mg) (Crinone^®^) was used.

### a. Embryo quality and transfer day

In 42.2% of the cases, at least one embryo of six to eight cells was transferred, with pattern A. Two to four quality A cells were found in 24.2% and in 6.4% of A embryos with 5, 9, 10, or 12 cells. Pattern B with six to eight cells occurred in 4.5% of the cycles, two to four cells at 5.8%, and five cells at 0.5%. Blastocysts were transferred in 16.4% of the cases. Of these, 88.8% were expanded.

49.4% of the transfers occurred on D3 of embryonic evolution and 34.2% on D2. 16.4% of the transfer was in blastocysts (D5). Up to four embryos were transferred per cycle, according to the patient’s age and following the guidance of the Federal Council of Medicine (CFM) in force at the time (Brazil, 2017). In 17.6%, a single embryo was transferred. Two embryos were implanted in the majority of cases (61.8%), three in 18.2% of cycles, and four embryos in 2.4% of cases.

### b. P4 and PRL dosages between pregnant and non-pregnant women

Table 1 shows the hormonal serum values between pregnant and non-pregnant women. A significant difference was observed in the P4 concentrations. Values above 32.1 ng/ml were associated with pregnancy. Regarding the PRL, no significance was observed.

**Table 1:** Progesterone and prolactin in pregnant and non-pregnant women (n=330).

Variable (ng/ml)	Pregnant (n=154)	Non-Pregnant (n=176)	*p*
P4	32.1 (19.7 - 46)	26.2 (17,4 -39,8)	0.013
PRL	20.7 (15.5 -34.7)	21.3 (14,2 -30,8)	0.303

Described by median and IIQ (1^st^ quartile - 3^rd^ quartile)

* Non-parametric Mann-Whitney test, *p*<0.05

Note: P4 - progesterone; PRL - prolactin; ng/ml - nanogram/milliliter

**Figure 1:**
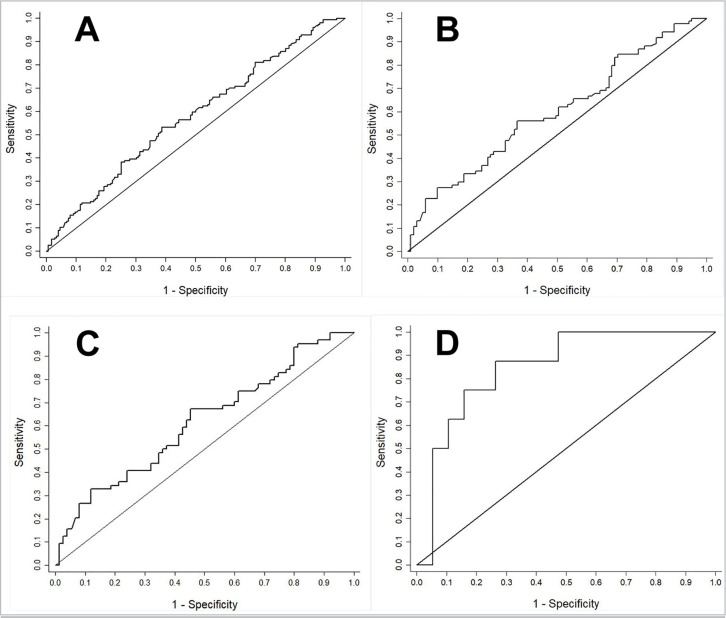
A. Association ROC Curve between progesterone (P4) serum assessment values and pregnancy B. **ROC curve of association between pregnancy and serum dosage of P4 in cycles with antagonist regardless of progesterone for luteal phase support** C. **ROC curve of association between pregnancy and serum dosage of P4 in cycles with antagonist and injectable progesterone use** D. **ROC curve of association between pregnancy and serum dosage of p4 in cycles without any blockage.**

A ROC curve was created, correlating progesterone and pregnancy (Figure 1a), with a sensitivity of 53.2% and specificity of 61.4%. The cut-off point established was 30.5 ng/ml.

### c. Serum concentrations of P4, PRL, type of pituitary blockage, and pregnancy

Due to the statistical relevance of P4, the sample was evaluated by the type of blockage and supplementation used and whether pregnancy was achieved or not.

#### Agonist use

Table 2 shows that no significant difference in age, number of embryos transferred, endometrial thickness, or association between P4, PRL, and pregnancy was observed. The serum P4 concentrations did not show differences between pregnant and non-pregnant women according to the type of progesterone administered. As shown in Table 3, PRL was found to be associated with pregnancy in users of Crinone^®^ when the dose reached 37.8 ng/ml

**Table 2:** Progesterone and prolactin between pregnant and non-pregnant women by the type of pituitary blockage (n=330).

Agonist (n=118)					Antagonist (n=185)			No Blockage (n=27)		
		**Pregnant**		*p* ^*^	**Pregnant**		*p* ^*^	**Pregnant**		*p* ^*^
		**Yes (n=62)**	**No (n=56)**		**Yes (n=84)**	**No (n=101)**		**Yes (n=8)**	**No (n=19)**	
Age (years)		34.3±3.5	35.4±3.6	0.123	34.8±4.0	36.49±4.3	0.008			
Endometrium (mm)		9.5±1.6	9.9±1.5	0.126	9.0±1.7	8.8±1.4	0.519	8.3±1.4	8.5±1.0	0.626
Number of	1-2	50 (80.6)	41 (73.2)	0.385	66 (78.8)	83 (82.2)	0.579	7 (87.5)	15 (78.9)	0.2729
Embryos	3-4	12 (19.4)	15 (26.8)		18 (21.4)	18 (17.8)		1 (12.5)	4 (21.1)	
P4 ng/ml		29.3 (19 - 43.2)	31.13 (18.4 - 44.9)	0.998	32.6 (19.7 - 55.38)	26.6 (16.8 - 41)	0.021	38.7 (31.6 - 50.3)	22.6 (14.1 - 28.1)	**0.004**
PRL ng/ml		24.7 (16.9 -35.4)	28.5 (19.2 - 35.9)	0.724	20.2 (14.30 - 33.2)	20.10 (13.8 - 29.6)	0.395	17.6 (16 - 19.4)	20.0 (14.3 - 25.2)	0.584

Described by median and IIQ (1^st^ quartile - 3^rd^ quartile)

* Non-parametric Mann-Whitney test, *p*<0.05

Note: P4 - progesterone; PRL - prolactin; ng/ml - nanogram/milliliter

**Table 3:** Progesterone and prolactin according to the type of blockage and to the progesterone used (n=330).

Blockage Type	Variable	Type of P4 used	Pregnant				*p* ^*^
			Yes		No		
Agonist (n=118)				n=62		n=56	
	P4 (ng/ml)	Injection USP^®^	31 (22 - 44.3)	51	36.6 (21 - 48.5)	42	0.378
		Utrogestan^®^	25.2 (16 - 45)	6	17.0 (10.3 - 21.9)	5	0.429
		Crinone^®^	11.5 (11.2 - 15.30)	5	18.1 (5.5 - 19.9)	9	1
	PRL (ng/ml)	Injection USP^®^	22 (16 - 36.4)	51	27.90 (18 - 36)	42	0.374
		Utrogestan^®^	24.4 (19.9 - 40.4)	6	18.3 (11 - 28.4)	5	0.428
		Crinone^®^	37.80 (25.5 - 51.2)	5	18 (13.7 - 25.6)	9	**0.029**
Antagonist (n=185)				n=84		n=101	***p*** ^*^
	P4 (ng/ml)	Injection USP^®^	37.8 (24.7 - 58.7)	64	29.8 (21.2 - 44.9)	75	**0.021**
		Utrogestan^®^	16.6 (14.5 - 19.7)	13	16.8 (11.7 - 25.8)	16	0.948
		Crinone^®^	19.8 (12.2 - 58.7)	7	13.0 (8.2 - 22)	10	0.109
	PRL (ng/ml)	Injection USP^®^	21.6 (14.9 - 36.1)	64	20.3 (14 - 31)	75	0.350
		Utrogestan^®^	25.6 (10.4 - 28.5)	13	21.0 (13.6 - 24.5)	16	0.503
		Crinone^®^	15.0 (9.1 - 17.9)	7	21.0 (13.6 - 24.5)	10	0.536
No Blockage (n=27)				n=8		n=19	
	P4 (ng/ml)	Injection USP^®^	50.3 (34.1 - 65.6)	4	25.8 (14.1 - 37.8)	11	0.104
		Utrogestan^®^	27.0	1	20.5 (17.4 - 25.4)	4	-
		Crinone^®^	37.9 (36.2 - 39.5)	3	18.1 (9.7 - 26.3)	4	-
	PRL (ng/ml)	Injection USP^®^	17.6 (17 - 19.3)	4	21.8 (18.3 - 25.2)	11	0.177
		Utrogestan^®^	18.0	1	16.1 (14.5 - 20.4)	4	-
		Crinone^®^	15.4 (14.9 - 29.4)	3	21.5 (13.5 - 28.7)	4	0.4161

Results described by median and IIQ (1^st^ quartile - 3^rd^ quartile)

* Non-parametric Mann-Whitney test. *p*<0.05

Note: P4 - progesterone; PRL - prolactin; ng/ml - nanogram per milliliter.

#### Antagonist use

Table 2 shows the association between age, progesterone dosage, and pregnancy in this group. Women aged 34.8 years had more pregnancies. The median of P4 in pregnant women was 32.6 ng/ml and 26.6 ng/ml in non-pregnant women. Figure 1b shows the ROC curve, with a sensitivity of 56.0% and specificity of 63.4% for pregnancy. The cut-off point was 30.5 ng/ml for pregnancy. Table 3 shows that antagonist and injectable progesterone were associated with pregnancy, when the median of P4 was 37.8 ng/ml (*p*=0.021). However, the PRL showed no relevance. The ROC curve showed a sensitivity of 67.2% and a specificity of 54.7% (Figure 1c) with a cut-off point of 30.5 ng/ml.

#### No pituitary blockage use

Table 2 shows the association of P4 and pregnancy, with a median of 38.7 ng/ml in pregnant women and 22.6 ng/ml in non-pregnant women. However, the PRL was not significant. The ROC curve showed a sensitivity of 87.5% and a specificity of 73.7% (Figure 1d) and a cut-off point of 26.7 ng/ml.

### d. Evaluation with logistic regression

#### Univariate analysis

Quantitative data on age, endometrial thickness, and P4 and PRL between pregnant and non-pregnant women (Table 4) entered the analysis. Age and P4 were significant, regardless of the type of blockage or supplementation (*p*=0.001 and *p*=0.021, respectively). More pregnancies in women under 34.7 years of age were observed, and the median P4 was over 31.9ng/ml.

**Table 4:** Univariated analysis of quantitative and qualitative variables.

	Variable		Pregnant		*p* ^*^	OR (IC95%)
			Yes	No		
Quantitative	Age (Years)		34.7±3.8	36.2±4.3	**0.001**	0.91 (0.86 - 0.96)
	Endometrial thickness (mm)		9.1±1.6	9.1±1.5	0.828	1.00 (0.88 - 1.15)
	P4 (ng/ml)		31.9 (19.5 - 46.1)	26.2 (17.2 - 40.2)	**0.021**	1.01 (1.002 - 1.02)
	PRL (ng/ml)		20.7 (15.6 - 34.8)	21.3 (14.2 - 30.9)	0.266	1.01 (0.99 - 1.02)
Qualitative			n	Pregnant n (%)	*p* ^*^	OR (IC95%)
	Blockage	None	27	8 (29.6%)		
		Antagonist	188	87 (46.3%)	0.127	1.97 (0.82 - 4.74)
		Agonist	115	59 (51.3%)	**0.036**	2.63 (1.07 - 6.48)
	Number of embryos	1 or 2	262	123 (47%)		
		3 or 4	68	31 (45.6%)	0.841	0.95 (0.55 - 1.62)
	Type of progesterone	Crinone	38	15 (39.5%)		
		Utrogestan	45	20 (44.4%)	0.648	1.23 (0.51 - 2.95)
		Progesterone	247	119 (48.2%)	0.319	1.43 (0.71 - 2.86)

Result described by mean ± standard deviation or median (1^st^ quartile - 3^rd^ quartile)

* Logistic Regression Model and Wald test. *p*<0.05

Note: P4 - progesterone; PRL-prolactin; ng/ml - nanogram / milliliter; mm - millimeters

The qualitative variables were blockage, number of embryos transferred, and type of P4 used. A correlation between agonist blocking and pregnancy was found (*p*=0.036).

#### Multivariate analysis

For multivariate analysis, age, P4, and type of pituitary block, which had statistical significance in the univariate analysis, were correlated with pregnancy. The first two criteria showed statistical significance. Age showed an odds ratio (OR) of 0.91 and a 95% confidence interval (CI) of 0.86-0.97 (*p*=0.002). For P4, we observed an OR of 1.01 and a 95% CI of 1.0-1.02 (*p*=0.027). As for pituitary blockage, no statistical significance was observed (*p*=0.204 for blockade with antagonists and *p*=0.087 for agonists).

## DISCUSSION

*In vitro* fertilization is a treatment with several particularities. In addition to a good embryo, a favorable endometrial and hormonal environment is essential. Hormone dosages in IVF allow for adjustments in treatment or better expectations in achieving pregnancy. According to the findings of this study, P4 dosages greater than 32.1 ng/mL may be useful in predicting whether or not pregnancy is achieved. Further studies are needed to assess whether adjustments in progesterone supplementation after embryo transfer would be effective in promoting pregnancy.

Despite the importance of P4 and PRL, only a few studies have investigated their serum concentrations after embryo placement. The choice of D9 after oocyte retrieval, between transfer and pregnancy test, was made to offer the support that IVF demands and maintain close contact with the patient undergoing treatment. Elevated levels of P4 were observed in greater numbers in pregnant women at this study. In the literature, serum progesterone above 1.5 ng/ml on the day of the oocyte trigger suggests early luteinization, indicating embryo freezing and transfer to another cycle to potentiate the result (Panaino *et al*., 2017). A P4 dosage of 10 ng/ml on D4 after the trigger in frozen embryo transfer provides better pregnancy rates (Gaggiotti-Marre *et al*., 2020).

The mean age of patients in this study was 35 years, which was lower than that reported in Latin America (37.2 years) (Zegers-Hochschild *et al*., 2022). Female age is one of the main factors that influence fertility, as it is directly linked to oocyte quality, which declines between 25 and 30 years of age and increases over time (Vander Borght & Wyns, 2018).

Endometriosis was found to be the main cause of infertility, which is present in 1/4 of the cases, contrary to the 10% described in the literature (de Ziegler *et al*., 2016). It was followed by male factor and ovarian failure, the latter being present in 32% of IVF cases in the United States (Pastore *et al*., 2018). The difference observed may be due to the service where the research was conducted, which has gynecological surgeons with a focus on video laparoscopy.

Endometrial thickness was similar to that observed in the literature (Kasius *et al*., 2014). The endometrium also needs a trilaminar appearance at the time of transfer to maximize the chances of success.

In this study, half of the cycles occurred with antagonists, which is used for its effectiveness and practicality to start cycles (Al-Inany *et al.,* 2016). When studying P4 in IVF, one should consider the type of pituitary blockage. Performed to inhibit the LH surge, it prevents ovulation before oocyte retrieval but interferes in the formation of P4 by the corpus luteum. LDL-cholesterol, the raw material of this hormone, needs LH to enter the mitochondria and initiate P4 production. By inhibiting LH, formation is impaired. Thus, we have several ineffective corpora lutea, leading to the need for supplementation (Taylor *et al*., 2019b). Agonist blockage acts on this axis for up to 10 days after the last application. The antagonist, for 24 hours (Fatemi, 2009). In unblocked cycles, increased ovarian steroid hormones do this job.

Luteal phase supplementation in this study was performed with IM P4, vaginal gel, or micronized natural P4. At the center that hosted the study, IM was the preferred route, which was used in 3/4 of the cases. It shows excellent absorption, but its application is painful and requires assistance to be administered. The used dose of micronized natural P4 requires the insertion of four vaginal eggs within 24 hours. However, it can ooze through the vulva and can be uncomfortable for the patient. The vaginal gel has better adherence to the vaginal fundus without this inconvenience.

A quality cleaved embryos were obtained in 74% of the cycles. In 1/6 of the cases, good blastocysts were found. The choice of treatments with good quality embryos was based on the analysis of the cycles with the potential for a positive result, thus being able to interpret the data between good cycles, hormonal dosages, and positive betas. Thus, the high rate was observed as part of the inclusion criteria. The better the embryonic quality, the higher the success rate; this data was also linked to age, as oocytes from young women tend to generate better embryos.

Approximately half of the transfers were performed on D3 and only 14.2% on blastocysts. It was common in the institution to transfer on D2 and D3, a fact that has changed in recent years with regard to blastocysts due to the improvement in embryonic cleavage rates. In almost half of the cycles, one to two embryos were transferred, respecting the orientation of the CFM.

When analyzing P4 and PRL collected on D9, regardless of supplementation, a higher pregnancy rate was observed when P4 was above 32.1 ng/ml. Moreover, PRL showed no significance.

Regarding the pituitary blockage, agonist cycles did not show significant differences regardless of the type of supplementation. For PRL, values of over 37.8 ng/ml in the agonist and Crinone^®^ combination were associated with pregnancy. However, the result should be interpreted with caution due to the small number of cases.

Agonist blockage is longer-lasting. Thus, the initial expectation was that high P4 values would indicate better hormone replacement and would occur in pregnant women, a fact that was not observed. In IM supplementation, the highest values were found in those who did not get pregnant. Using Utrogestan^®^, the opposite occurred in pregnant women with higher P4 values. Further studies are needed to assess whether this is the best agonist supplementation.

Cycles with an antagonist were associated with age, P4, and pregnancy. Moreover, P4 values collected on D9 above 32.6 ng/ml were associated with pregnancy. The antagonist and injectable progesterone combination showed more pregnancies if the values were above 37.8 ng/ml. The serum concentrations of PRL were not significant.

In blockage with antagonist, elevated serum P4 values were observed in pregnant women as expected, and the ROC curve cut-off value was 30.5 ng/ml. The values of IM supplementation were also expected, as the antagonist blocks the axis for a shorter time, continuing the function of the corpus luteum more quickly.

Cases without block and P4 above 38.7 ng/ml were associated with pregnancies. Despite the blockage occurring due to high steroid levels and not due to pituitary action, the high P4 was consistent with the initial expectation. However, the other analyses showed no significant findings. The ROC curve showed its best performance with a cut-off point of 26.7 ng/ml. An assumption for the lower value of P4 in relation to the other curves in the study may be related to a less effective block and normal functioning of the formed corpus luteum. Thus, one possibility is that lower doses of P4 would already reach the level necessary for embryo fixation. Agonists tend to improve pregnancy rates in cycles compared with antagonists (Lambalk *et al*., 2017), a combination widely used for endometriosis due to the embryonic quality obtained (Xiao & Yu, 2021). However, no studies have compared agonists with non-blocking cycles.

The multivariate analysis considered age, P4, and block, assessing the significance of the first two. The data indicated that with each year of age, a 9% reduction was observed in the chance of getting pregnant, in relation to the basal rate, which is consistent with the findings of the literature (Vander Borght & Wyns, 2018). As for P4, in the present study, for each ng/ml more than P4, an increase of 1% in the probability of getting pregnant was observed.

In the present study, PRL showed no significant association with outcomes or variables. Other authors have reported that increased serum concentrations of PRL lead to poorer quality embryos and miscarriages (Kim *et al*., 2018).

However, this study has some limitations related to its retrospective design. It was not confirmed whether progesterone supplementation was performed. It is known that patients undergoing IVF are highly motivated to use the medication correctly. Additionally, all patients were treated by members of the clinical staff of the same team and followed the same protocols, ensuring consistency in the treatment process.

The cut-off points found in this work are unprecedented for analysis of D9 after oocyte capture. The purpose of this study is not to replace the beta-hCG test but to provide subsidies that can help the attending physician follow up his patient in the week before the pregnancy diagnosis. IVF treatments go along with great anxiety, often being exhausting from a psychological point of view. Thus, monitoring treatments brings security to patients who receive assistance at each stage of the journey.

## CONCLUSION

The measurement of serum P4 concentrations performed on D9 after oocyte retrieval was useful in predicting success in the IVF treatment cycle. Serum P4 levels above 32.1 ng/ml collected on D9 after oocyte pick-up was associated with pregnancy. The value was influenced by the type of pituitary blockage and the route of administration of the supplement used.

Serum concentrations of PRL did not show statistical significance in any of the scenarios evaluated.
